# OXA-23 β-Lactamase Overexpression in Acinetobacter baumannii Drives Physiological Changes Resulting in New Genetic Vulnerabilities

**DOI:** 10.1128/mBio.03137-21

**Published:** 2021-12-07

**Authors:** Jennifer M. Colquhoun, Marjan Farokhyfar, Anna R. Hutcheson, Alexander Anderson, Christopher R. Bethel, Robert A. Bonomo, Anthony J. Clarke, Philip N. Rather

**Affiliations:** a Department of Microbiology and Immunology, Emory University, Atlanta, Georgia, USA; b Emory Antibiotic Resistance Center, Emory University, Atlanta, Georgia, USA; c Research Service, Atlanta VA Medical Center, Decatur, Georgia, USA; d Department of Molecular and Cellular Biology, University of Guelph, Guelph, Ontario, Canada; e Research Service, Louis Stokes Cleveland Department of Veterans Affairs, Cleveland, Ohio, USA; f Department of Biochemistry, Case Western Reserve University, Cleveland, Ohio, USA; g Department of Medicine, Case Western Reserve University, Cleveland, Ohio, USA; h Department of Molecular Biology and Microbiology, Case Western Reserve University, Cleveland, Ohio, USA; i Department of Pharmacology, Case Western Reserve University, Cleveland, Ohio, USA; j Department of Proteomics and Bioinformatics, Case Western Reserve University, Cleveland, Ohio, USA; k Department of Chemistry & Biochemistry, Wilfrid Laurier University, Waterloo, Ontario, Canada; University of Georgia

**Keywords:** *Acinetobacter*, beta-lactamases, peptidoglycan

## Abstract

β-Lactamase expression is the major mechanism of resistance to penicillins, cephalosporins, and carbapenems in the multidrug-resistant (MDR) bacterium Acinetobacter baumannii. In fact, stable high-level expression of at least one β-lactamase has been rapidly increasing and reported to occur in up to 98.5% of modern A. baumannii isolates recovered in the clinic. Moreover, the OXA-51 β-lactamase is universally present in the A. baumannii chromosome, suggesting it may have a cellular function beyond antibiotic resistance. However, the consequences associated with OXA β-lactamase overexpression on A. baumannii physiology are not well understood. Using peptidoglycan composition analysis, we show that overexpressing the OXA-23 β-lactamase in A. baumannii drives significant collateral changes with alterations consistent with increased amidase activity. Consequently, we predicted that these changes create new cellular vulnerabilities. As proof of principle, a small screen of random transposon insertions revealed three genes, where mutations resulted in a greater than 19-fold loss of viability when OXA-23 was overexpressed. The identified genes remained conditionally essential even when a catalytically inactive OXA-23 β-lactamase was overexpressed. In addition, we demonstrated a synergistic lethal relationship between OXA-23 overexpression and a CRISPR interference (CRISPRi) knockdown of the essential peptidoglycan synthesis enzyme MurA. Last, OXA-23 overexpression sensitized cells to two inhibitors of peptidoglycan synthesis, d-cycloserine and fosfomycin. Our results highlight the impact of OXA-23 hyperexpression on peptidoglycan integrity and reveal new genetic vulnerabilities, which may represent novel targets for antimicrobial agents specific to MDR A. baumannii and other OXA β-lactamase-overexpressing *Enterobacteriaceae*, while having no impact on the normal flora.

## INTRODUCTION

Multidrug-resistant (MDR) Acinetobacter baumannii is a significant global health problem responsible for approximately two million infections and one hundred thousand deaths annually worldwide (1). Making this burden more troublesome is that few antibiotics remain effective against this pathogen. Simply put, it is critical we acquire a deeper understanding of the mechanisms responsible for A. baumannii cellular adaptation, antibiotic resistance, and pathogenesis to aid in the development of novel therapeutic treatments.

The most widespread and threatening mechanism of antibiotic resistance in MDR A. baumannii is the production of different β-lactamase enzymes. The pathogen utilizes several intrinsic and acquired β-lactamase enzymes to inactivate β-lactams and survive treatment. More specifically, A. baumannii universally encodes two β-lactamases on its chromosome, *bla*_ADC_ (AmpC) and *bla*_OXA-51_, suggesting they may have yet-to-be-identified cellular housekeeping functions (2, 3). In fact, β-lactamases are believed to have evolved from penicillin binding proteins (PBPs) based on sequence alignments and comparisons of tertiary structures and serine active sites (4). PBPs are a collection of enzymes that function in peptidoglycan (PG) synthesis with their primary activities being transglycosylation to incorporate PG precursors and transpeptidation for the cross-linking of adjacent PG pentapeptide side chains essential for structural integrity and cell shape (5, 6). Although β-lactamase evolution studies have shown the enzymes have lost their d,d-carboxypeptidase activity in favor of increased β-lactam hydrolysis rates, a small but detectable level of *in vitro* d,d-peptide hydrolysis has been maintained in all β-lactamase classes (7–9). These studies indicate that β-lactamases may still bind to and act upon a PG substrate, although to a much lesser extent than their PBP relatives.

While β-lactamase contribution to antibiotic resistance has been widely investigated, few studies have explored the cellular impact associated with β-lactamase overexpression. Moreover, many of these studies present conflicting results on the phenotypic or fitness costs associated with different β-lactamases and/or bacterial species. For example, Fernandez and colleagues showed that overexpression of class D β-lactamases (OXA-10-like and OXA-24) and one class A β-lactamase representative (SFO-1) displayed alterations in PG composition and reductions in overall cross-linking, biofilm formation, and *in vitro* and *in vivo* competition assays despite no changes in morphology or growth rates in Escherichia coli (10). Gallant and colleagues showed that class A β-lactamase TEM-1 and class D β-lactamase OXA-3 overexpression, but not class A β-lactamase BlaS or class C β-lactamase AmpC, resulted in biofilm defects with no impact on growth rate in both Pseudomonas aeruginosa and E. coli (11). Further, this study demonstrated that β-lactamase active site inactivation of TEM-1, but not OXA-3, restored wild-type biofilm formation levels. Finally, Morosini and colleagues showed that AmpC hyperexpression in Salmonella enterica serotype Typhimurium was detrimental to growth rate and cell invasion (12), whereas recent studies of P. aeruginosa have shown that hyperexpression of AmpC does not impact growth rate (13, 14). However, these same studies showed that simultaneous blocking of PG recycling, by triple *ampD*-type amidase or *ampG* knockout, with AmpC derepression resulted in growth rate, motility, virulence, and cytotoxicity defects. Moreover, these studies revealed that AmpC overexpression was necessary but not sufficient for the defects observed in the *ampD* triple mutant, suggesting that AmpC overexpression generates cellular damage that requires additional mutations to be observed.

The goals of this study were to understand the cellular impact of β-lactamase overexpression on bacterial physiology by determining the collateral effects of *bla*_OXA-23_ β-lactamase overexpression in A. baumannii and identifying genes that become conditionally essential when OXA-23 is overexpressed. This β-lactamase was chosen because it is the most frequently encountered OXA enzyme in A. baumannii (15–17). We report substantial alterations in peptidoglycan structure when the OXA-23 β-lactamase was overexpressed and identify three genes that become conditionally essential when OXA-23 was expressed. Several of these genes are predicted to influence peptidoglycan structure, and we demonstrate that moderate inhibition of the first step in peptidoglycan synthesis catalyzed by MurA results in lethality when the OXA-23 β-lactamase is overexpressed. Overall, our results support that OXA-23 β-lactamase maintains some of its ancestral PBP function that, when overexpressed, achieves antibiotic resistance but at the cost of normal peptidoglycan synthesis in A. baumannii.

## RESULTS

### Characterization of the effects of OXA-23 overexpression in A. baumannii.

As a starting point to elucidate the effects of OXA-23 β-lactamase overexpression in A. baumannii, we set out to evaluate transcriptional and peptidoglycan composition changes induced by OXA-23 hyperexpression. First, we generated an ATCC 17978 strain containing an isopropyl-β-d-1-thiogalactopyranoside (IPTG)-inducible *tac_p_*-*bla*_OXA-23_ gene within a single copy Tn*7* derivative and confirmed that this strain displayed a significant increase in ampicillin and imipenem resistance upon OXA-23 overexpression (Table 1). Indeed, we observed >8-fold and 128-fold increase in ampicillin and imipenem MICs, respectively, when the *bla*_OXA-23_ gene was induced with IPTG, whereas the MICs were equivalent to those of the wild-type parent under noninducing conditions. Further, there was no change in the growth kinetics of A. baumannii under control and OXA-23-overexpressing conditions (see Fig. S1 in the supplemental material). Using comparative transcriptome sequencing (RNA-Seq) analysis, we estimate that expression from the IPTG-inducible *tac_p_*-*bla*_OXA-23_ is approximately fourfold higher in strain ATCC 17978 than the levels where an IS*Aba1* drives a single copy *bla*_OXA-23_ in the chromosome of strain AB5075 (data not shown).

**TABLE 1:** MICs of A. baumannii IPTG-inducible OXA-23 β-lactamase strains

Strain	Ampicillin MIC (μg/ml)		Imipenem MIC (μg/ml)		Fold increase^*a*^
	Control	With IPTG	Control	With IPTG	
ATCC 17978 (WT^*b*^)	32	32	0.25	0.25	ND
Tn*7*::*bla*_OXA-23_	32	>256	0.25	32	80.9 ± 5.4
Tn*7*::*bla*_OXA-23(S79A)_	32	32	0.25	0.25	ND

a The reported value represents the fold increase in the presence of 5 mM IPTG determined by qRT-PCR. ND, not determined.

b WT, wild type.

10.1128/mBio.03137-21.5 FIG S1 Growth curves of ATCC 17978 Tn*7*::*bla*_OXA-23_ in LB with and without 5 mM IPTG. Three independent colonies were picked directly into 1 ml LB and grown overnight at 28°C. Overnight cultures were diluted to an OD_600_ of 0.05 in 2 ml LB and supplemented with 5 mM IPTG. Cultures were grown for 8 h with shaking at 37°C with OD_600_ monitored every 30 min. Download FIG S1, TIF file, 1.8 MB. Copyright © 2021 Colquhoun et al. 2021 Colquhoun et al. https://creativecommons.org/licenses/by/4.0/ This content is distributed under the terms of the Creative Commons Attribution 4.0 International license.

Next, the impact of OXA-23 overexpression on the A. baumannii global transcriptome was determined by RNA-Seq. Besides the 99.73-fold increase in *bla*_OXA-23_ transcript upon IPTG induction, no other transcripts showed more than a twofold change compared to the wild-type control (see Table S1 in the supplemental material). The increase in *bla*_OXA-23_ expression with IPTG induction was confirmed by quantitative reverse transcription-PCR (qRT-PCR) in three independent cultures, where we saw an average fold increase of 80.9 ± 5.4 (Table 1).

10.1128/mBio.03137-21.1 TABLE S1 OXA-23 overexpression RNA-Seq data. Download Table S1, XLSX file, 0.4 MB. Copyright © 2021 Colquhoun et al. 2021 Colquhoun et al. https://creativecommons.org/licenses/by/4.0/ This content is distributed under the terms of the Creative Commons Attribution 4.0 International license.

**FIG 1:**
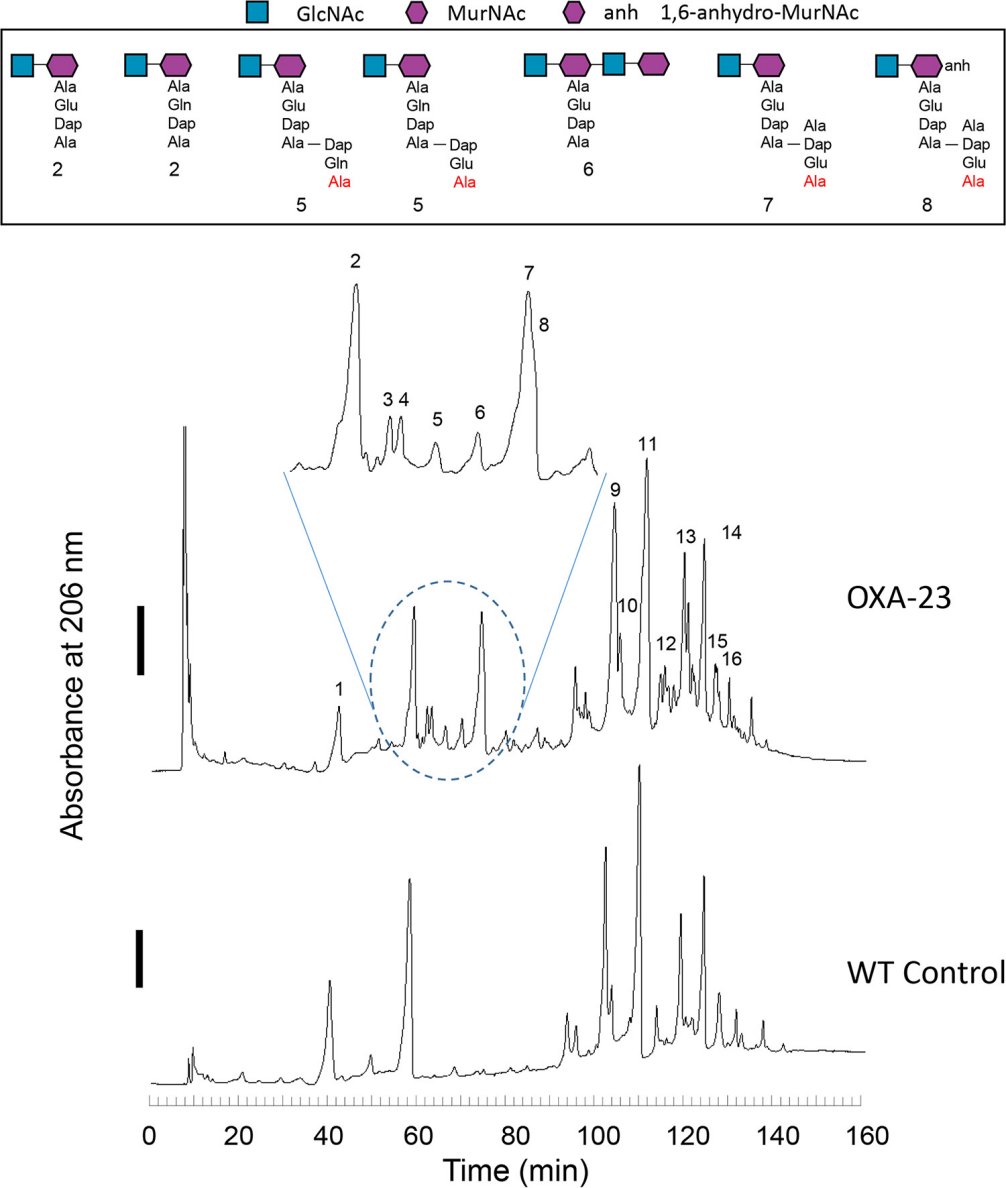
Representative chromatograms of the peptidoglycan composition and percent cross-linking of wild-type A. baumannii ATCC 17978 and ATCC 17978 overexpressing OXA-23. Overnight cultures were diluted to an OD_600_ of 0.02 and supplemented with 2 mM IPTG. Cultures were grown shaking at 37°C to an OD_600_ of 0.8, and then cells were pelleted by centrifugation. Whole peptidoglycan was purified and processed as described by Schaub and Dillard (36). Peptidoglycan was subjected to high performance liquid chromatography (HPLC) separation and LC-MS identification in triplicate. The solid bars to the left of the chromatograms denote 0.25 AU (absorbance units). Muropeptides associated with the identified fractions are listed in Table S2 in the supplemental material.

We next assessed alterations to peptidoglycan (PG) composition due to OXA-23 overexpression (Fig. 1). Muropeptide composition of isolated and digested PG sacculi was assessed using reverse-phase high-pressure liquid chromatography (RP-HPLC) and liquid chromatography-quadrupole time of flight mass spectrometry (LC–Q-TOF-MS) analyses. The muropeptide profiles of three technical replicates of wild-type- and OXA-23-overexpression-derived PG showed differences in the fractions at various retention times (Fig. 1, blue dashed circle). LC-MS analysis of the differential abundance peaks identified in the OXA-23 overexpression PG revealed an increase in tetrasaccharide (GlcNAc-MurNAc-GlcNAc-MurNAc) with only one stem peptide (peak 6) and disaccharides with two cross-linked peptide stems (peaks 5, 7, and 8), which are known products of amidase activity (18, 19). Table S2 provides the muropeptide composition of each strain. Further, OXA-23 overexpression resulted in a significant increase in the percent cross-linking compared to the control (46.1% ± 0.330% versus 40.7% ± 0.102%). Overall, these results suggest that OXA-23 overexpression confers β-lactam resistance at the expense of collateral PG composition changes. The increased cross-linking in the OXA-23-overexpressing strain may represent a compensatory response to counteract the increased amidase activity.

10.1128/mBio.03137-21.2 TABLE S2 Identification of muropeptides in mutanolysin digests from wild-type and OXA-23-expressing Acinetobacter baumannii ATCC 17978. Download Table S2, DOCX file, 0.10 MB. Copyright © 2021 Colquhoun et al. 2021 Colquhoun et al. https://creativecommons.org/licenses/by/4.0/ This content is distributed under the terms of the Creative Commons Attribution 4.0 International license.

To follow up on the increased PG amidase activity associated with cellular OXA-23 overexpression, we sought to demonstrate that purified OXA-23 enzyme directly cleaved purified PG sacculi using a Remazol brilliant blue dye release assay (20). Although the lysozyme positive control displayed robust PG cleavage activity, we were unable to detect OXA-23 PG amidase activity alone, supplemented with ion or metal cofactors, under various reaction pHs, or with the addition of A. baumannii whole-cell lysate (Fig. S2).

10.1128/mBio.03137-21.6 FIG S2 Purified OXA-23 *in vitro* enzymatic activity. (A) Nitrocefin cleavage kinetics of 2.5 ng OXA-23 performed in triplicate to confirm β-lactamase activity. (B) Remazol Brilliant Blue R (RBB)-stained A. baumannii peptidoglycan (PG) cleavage of purified OXA-23. Each reaction mixture was incubated at 37°C for 24 h, and cleavage activity was determined by solubilized blue RBB-stained muropeptides measured at 595 nm. The following reaction conditions were tested: 4 μM lysozyme (positive), RBB-stained PG alone (negative), 20 μM OXA-23, 40 μM OXA-23, 80 μM OXA-23, 20 μM OXA-23 supplemented with 4 mM MgCl_2_, ZnCl_2_, MnCl_2_, NiCl_2_, CoCl_2_, CaCl_2_, or FeCl_2_, 20 μM OXA-23 in PBS adjusted to pH 5, 6, 7, 8, or 9, or 20 μM OXA-23 supplemented with 7.5, 15, 30, or 60 μl A. baumannii whole-cell lysate. Download FIG S2, TIF file, 1.8 MB. Copyright © 2021 Colquhoun et al. 2021 Colquhoun et al. https://creativecommons.org/licenses/by/4.0/ This content is distributed under the terms of the Creative Commons Attribution 4.0 International license.

### Identification and characterization of genes that become conditionally essential during OXA-23 overexpression in A. baumannii.

Due to the observed changes in PG composition upon OXA-23 overexpression in A. baumannii, we hypothesized that this collateral damage may create new genetic vulnerabilities, where genes that are normally dispensable will become essential when the OXA-23 β-lactamase is overexpressed. A proof of principle screen of approximately 2,000 EZ-Tn*5* <KAN-2> transposon insertions in strain ATCC 17978 identified insertions that displayed a significant loss of viability upon OXA-23 overexpression with IPTG. We identified five unique insertions mapping to three genes that encoded the cell division protein ZipA, a predicted M23/M37 family peptidase (A1S_1185) and a putative glutathione *S*-transferase (A1S_0408). For *zipA*, the transposon inserted at a location where it would disrupt amino acid 98 of the 345-amino-acid protein. For *A1S_1185*, the insertion disrupted amino acid 101 of the 268-amino-acid protein and for *A1S_0408*, the insertion disrupted amino acid 171 of the 201-amino-acid protein.

**FIG 2:**
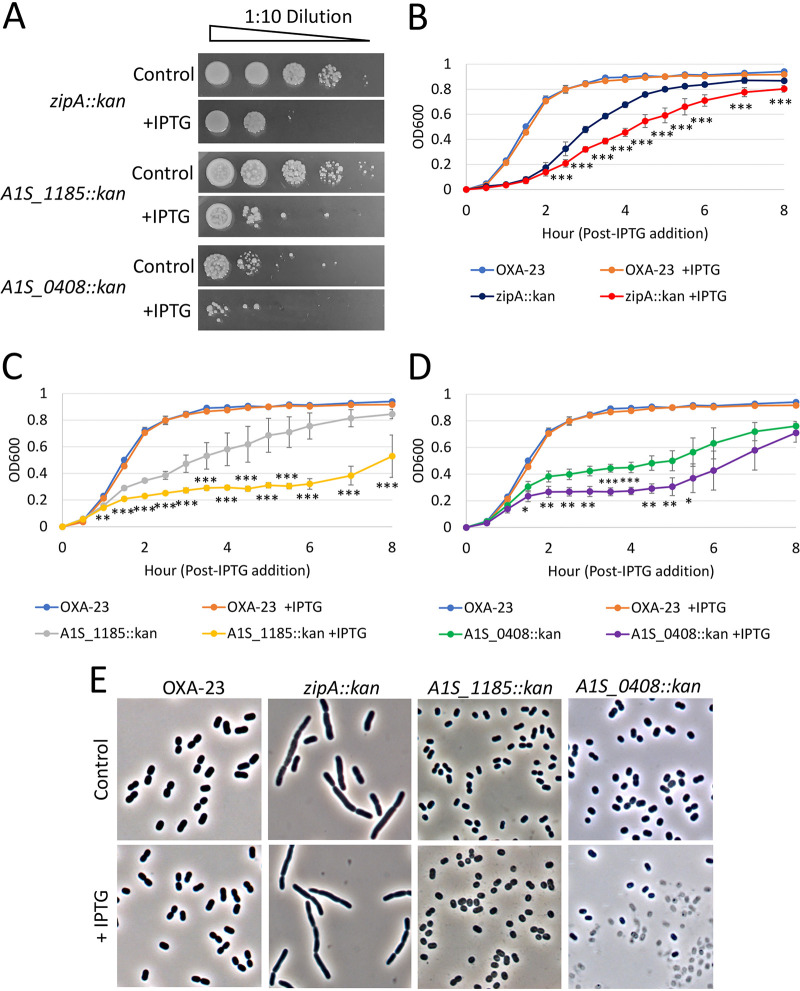
Characterization of genes required for survival when OXA-23 is overexpressed. (A) Strain ATCC 17978 Tn*7*::*bla*_OXA-23_ with indicated transposon insertions was spotted on LB agar supplemented with 30 μg/ml kanamycin (Control) and 5 mM IPTG to induce OXA overexpression (+IPTG). (B to D) Growth curves of ATCC 17978 Tn*7*::*bla*_OXA-23_ control and ATCC 17978 Tn7::*bla*_OXA-23_ transposon insertions in LB with and without 5 mM IPTG. Six independent transformants were picked directly into 1 ml LB plus 30 μg/ml kanamycin and grown overnight at 28°C. Overnight cultures were diluted to an OD_600_ of 0.05 in 2 ml LB and supplemented with 5 mM IPTG. Cultures were grown for 8 h shaking at 37°C with OD_600_ monitored every 30 min. (E) Phase-contrast microscopy of ATCC 17978 Tn*7*::*bla*_OXA-23_ control and ATCC 17978 Tn*7*:: *bla*_OXA-23_ transposon insertions. Samples were collected after 3 h growth after IPTG addition as described above. Cell morphology was visualized using ×1,000 magnification on an Olympus BX51 microscope and imaged using a Lumenera Infinity2 camera. Statistical significance determined by paired two-tailed Student’s *t* test is indicated by asterisks as follows: *, *P* < 0.05; **, *P* < 0.01; ***, *P* < 0.001.

To better understand the impact of the identified genes that become conditionally essential alone and during OXA-23 overexpression, we evaluated each mutant’s viability, growth kinetics, and cell morphology under normal and inducing conditions (Fig. 2). Spot dilutions of each mutant on control and IPTG-containing plates confirmed our screening results, where each mutation resulted in loss of viability under inducing conditions compared to uninduced controls (Fig. 2A). To quantitate the reduction in viability, the percent survival of cells transformed with each mutation was determined in the presence and absence of IPTG. The *zipA*::*kan* mutation resulted a 19-fold reduction in viability on IPTG-containing plates, the *A1S_1185*::*kan* led to a 48-fold reduction and *A1S_0408*::*kan* resulted in a 25-fold reduction (Table 2). Transformation of each mutation into a strain containing an empty Tn*7* gave a similar number of transformants in the presence and absence of IPTG (data not shown). When evaluating growth kinetics, we found that each mutation alone significantly reduced growth rates (Fig. 2B to D), and the *zipA*::*kan* mutation also increased the time in lag phase (Fig. 2B). Moreover, OXA-23 overexpression in each mutant further reduced growth rates (Fig. 2B to D).

**TABLE 2:** Percent survival of mutants under WT and catalytically inactivated OXA-23 β-lactamase-overexpressing conditions

Mutant	% survival^*a*^		*P* value^*b*^
	Wild-type	Inactive (S79A)	
*zipA*::*kan*	5.3 ± 2.8	4.7 ± 3.2	0.9
*A1S_1185*::*kan*	2.1 ± 0.9	2.3 ± 1.9	0.8
*A1S_0408*::*kan*	4.0 ± 1.6	8.6 ± 2.3	0.07

a Values represent the averages of three to five biological replicates with standard deviations shown.

b Determined by paired two-tailed Student’s *t* test.

Using phase-contrast microscopy, *zipA*::*kan* mutant cells displayed elongated cells and chaining due to division defects (Fig. 2E), as previously published (21). Upon OXA-23 overexpression, *zipA*::*kan* cells displayed a slight increase in overall length, but these differences were not significant (3.68 ± 1.88 μm to 4.08 ± 2.19 μm; *P* = 0.086). Cells of the *A1S_1185*::*kan* mutant exhibited a smaller overall size and cell rounding compared to the control (Fig. 2E); however, *A1S_1185*::*kan* cells did not display significant changes to cell morphology upon OXA-23 overexpression. Conversely, *A1S_0408*::*kan* mutant cells showed a smaller overall size and cell rounding in addition to a high proportion of ghost cells upon OXA-23 overexpression (Fig. 2E). Altogether, our results suggest that mutations in the genes identified to become conditionally essential upon OXA-23 overexpression may exacerbate peptidoglycan synthesis defects, initially imposed by OXA-23 overexpression, thus leading to significant impacts on growth, morphology, and viability.

To confirm that the transposon insertions were responsible for the loss of viability upon OXA-23 overexpression, we conducted complementation experiments by directly introducing each transposon insertion via electroporation of genomic DNA from each mutant into a *bla*_OXA-23_-inducible strain with the respective wild-type gene on a plasmid or in a strain with vector control. The number of CFU was then determined on media with or without IPTG (Table 3). The conditional essential phenotype of the *zipA*::*kan* mutant was successfully complemented, showing that the ZipA protein is critical for viability during OXA-23 overexpression. The *A1S_1185*::*kan* mutation was not complemented by *A1S_1185* expression in *trans* (2.1-fold increase in survival; *P* = 0.06). Since the complementation was done in multicopy, we hypothesize that excess M23/M37 peptidase activity may also be detrimental to cell viability due to PG damage, much like OXA-23 overexpression, which may explain the partial complementation phenotype. Last, the *A1S_0408*::*kan* mutation was also not complemented by the cloned *A1S_0408* gene in *trans* (3.2-fold increase in survival; *P* = 0.26).

**TABLE 3:** Complementation of conditionally essential mutants during OXA-23 overexpression

Mutant	% survival on IPTG plates^*a*^		*P* value^*b*^
	Vector	Complement	
*zipA*::*kan*	3.4 ± 2.8	86.5 ± 8.4	0.007
*A1S_1185*::*kan*	2.2 ± 2.0	4.5 ± 2.3	0.06
*A1S_0408*::*kan*	2.6 ± 1.0	8.4 ± 6.5	0.26

a Values represent the averages of three biological replicates with standard deviations shown.

b Determined by paired two-tailed Student’s *t* test.

### Synthetic lethality does not require OXA-23 β-lactamase activity.

Since initial characterization of OXA-23 overexpression in A. baumannii cells revealed both β-lactam resistance and increased PG amidase activity, we determined whether β-lactamase function was necessary for the synthetic lethal phenotype of the above mutations (Table 2). To test this, we generated an IPTG-inducible *bla*_OXA-23_ mutant with the β-lactamase catalytic serine mutated to an alanine (OXA-23-S79A) and confirmed that OXA-23-S79A overexpression did not result in ampicillin or imipenem resistance (Table 1). Next, we evaluated the viability of our conditionally essential mutants under noninducing and inducing conditions in both the wild-type OXA-23 and catalytically inactive OXA-23-S79A overexpression strains by directly plating transformants from electroporations with genomic DNA from each mutant on kanamycin plates with and without IPTG and measuring CFU. Neither *zipA*::*kan*, *A1S_1185*::*kan* nor *A1S_0408*::*kan* displayed statistically significant differences in IPTG sensitivity between the wild-type or catalytically inactive OXA-23 overexpression strains, indicating that OXA-23 β-lactamase activity was not required for inducible lethality.

### OXA-23 overexpression confers synthetic lethality with CRISPRi knockdown or chemical inhibition of peptidoglycan synthesis.

**FIG 3:**
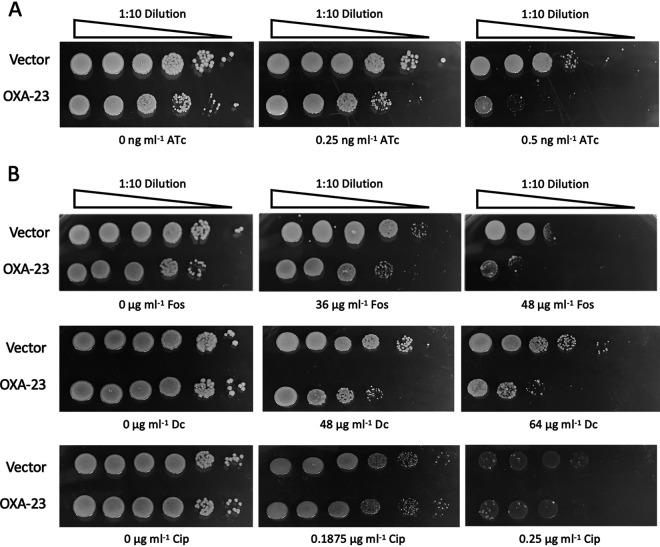
Synergistic lethality of OXA-23 overexpression and a CRISPRi *murA* knockdown or chemical inhibition of peptidoglycan synthesis enzymes. (A) Cells of ATCC 17978 Tn*7*::dCas9 pJE53::murA-sgRNA pQF1266-hyg (vector) or ATCC 17978 Tn*7*::dCas9 pJE53::murA-sgRNA pQF1266-hyg::*bla*_OXA-23_ were spotted on LB agar plates supplemented with 30 μg/ml KAN, 150 μg/ml HYG and 0, 0.25, or 0.5 ng ml^−1^ anhydrotetracycline (ATc) to induce *murA* sgRNA expression. OXA-23 β-lactamase and dCas9 genes are constitutively expressed in these strains. *murA* knockdown at 1 ng ml^−1^ ATc was lethal for both strains (data not shown). (B) Cells of ATCC 17978 Tn*7*::dCas9 pQF1266-hyg (vector) or ATCC 17978 Tn*7*::dCas9 pQF1266-hyg::*bla*_OXA-23_ were spotted on LB agar plates supplemented with 150 μg/ml HYG and 0, 3, 6, or 48 μg/ml fosfomycin (Fos; MurA inhibitor), 0, 48, or 64 μg ml^−1^ d-cycloserine (Dc), dual alanine racemase Alr and d-alanine:d-alanine ligase Ddl inhibitor) or 0, 0.1875, or 0.25 μg/ml ciprofloxacin (CIP; dual DNA topoisomerase and DNA gyrase inhibitor). OXA-23 β-lactamase and dCas9 genes are constitutively expressed in these strains. Bacterial growth completely inhibited for both strains at 72 μg/ml Fos, 128 μg/ml Dc, and 0.5 μg/ml CIP (data not shown).

Our data implied that OXA-23 overexpression disrupted normal A. baumannii PG synthesis or maintenance; therefore, we predicted that knockdown of an essential PG biosynthetic enzyme in combination with OXA-23 overexpression would result in a synergistic loss of viability. To test this, we generated and tested the viability of an anhydrotetracycline (ATc)-inducible CRISPR interference (CRISPRi)-mediated *murA* knockdown strain with a plasmid constitutively expressing high levels of OXA-23. The MurA enzyme, UDP-*N*-acetylglucosamine enolpyruvyl transferase, carries out the first step in peptidoglycan synthesis (22). We observed that OXA-23 overexpression in combination with 10.8-fold reduction in *murA* transcript resulted in a small colony phenotype and a 4.32 ± 1.18-fold reduction in cell viability compared to the *murA* knockdown alone at 0.25 ng ml^−1^ anhydrotetracycline (ATc) (Fig. 3A). To further support these results, we evaluated the sensitivity of A. baumannii overexpressing OXA-23 to fosfomycin, a known MurA inhibitor (Fig. 3B). Similarly, OXA-23 overexpression resulted in a 7.17 ± 2.97-fold reduction in cell viability compared to the vector control at subinhibitory fosfomycin (36 μg ml^−1^; 0.5× MIC).

To confirm that OXA-23 overexpression results in hypersensitivity specific to peptidoglycan synthesis inhibition, we evaluated the susceptibility of OXA-23-overexpressing cells to another peptidoglycan synthesis enzyme inhibitor, d-cycloserine, which inhibits both alanine racemase and d-alanine:d-alanine ligase (23) (Fig. 3B). OXA-23 overexpression resulted in a 65.19 ± 7.33-fold reduction in cell viability compared to the vector control at subinhibitory d-cycloserine (64 μg ml^−1^; 0.5× MIC). In addition, OXA-23-overexpressing cells displayed no change in sensitivity to ciprofloxacin, a DNA topoisomerase/DNA gyrase inhibitor, compared to the vector control (0.98 ± 0.45-fold reduction in cell viability at 0.1875 μg ml^−1^ [0.75× MIC]). These results indicate that fosfomycin and d-cycloserine hypersensitivity in OXA-23-overexpressing A. baumannii is not due to reduced membrane integrity (Fig. 3B). Overall, our data support that the collateral changes to PG composition imposed by OXA-23 overexpression potentiate the negative impact of reducing PG biosynthesis by specific genetic or chemical inhibition.

## DISCUSSION

The goals of this study were to elucidate the collateral effects associated with OXA-23 overexpression and uncover new genetic vulnerabilities associated with this overexpression in A. baumannii. Our work demonstrated that OXA-23 overexpression in A. baumannii drives β-lactam resistance and PG composition alterations without significant changes to the global transcriptome or growth rate. This finding is consistent with a previous publication that showed OXA-10 and OXA-24 overexpression exhibited PG composition changes without any impact on morphology and growth rate in Escherichia coli (10). Further, we identified three genes, *zipA* (encoding a cell division protein), *A1S_1185* (encoding a predicted M23/M37 family peptidase), and *A1S_0408* (encoding a putative glutathione *S*-transferase), that are normally dispensable for growth yet became essential during OXA-23 overexpression. Our results add to several publications that have shown a broad range of phenotypic changes associated with β-lactamase overexpression, or derepression, in other Gram-negative bacteria, including but not limited to alterations in growth rate, cell morphology, motility, biofilm formation, and virulence (10–14, 24).

To understand the mechanism behind OXA-23 overexpression’s physiological effects, we determined that the conditional essentiality of the above mutations did not require OXA-23 β-lactamase activity. Interestingly, another study observed that substitution of the catalytic serine in OXA-3 to render it inactive did not abolish the biofilm formation defect in Pseudomonas aeruginosa and E. coli when this β-lactamase was expressed (11). Additionally, we further demonstrated the detrimental effects of altering PG synthesis together with OXA-23 overexpression by showing a synergistic lethal relationship between OXA-23 hyperexpression and genetic or chemical inhibition of two PG synthesis enzymes. Similarly, recent studies have shown mutations that simultaneously block PG recycling and induce AmpC β-lactamase overexpression impair P. aeruginosa fitness and virulence and result in a significant reduction in total PG concentration per cell (13, 14).

On the basis of our results, we hypothesize that high levels of OXA-23 β-lactamase lead to increased PG amidase activity, which may directly impact PG integrity. Consequently, the collateral PG damage likely results in broad cellular stress responses. We predict that both the direct damage to PG as well as physiological stress due to OXA-23 overexpression results in certain genes becoming essential under the above conditions, but not in wild-type bacteria. Interestingly, the genes we identified as conditionally essential during OXA-23 overexpression have functions related to cell wall homeostasis. ZipA is a membrane-anchored protein that interacts with FtsZ and bifunctional PBPs to localize and stimulate preseptal peptidoglycan synthesis for cell division (25, 26). A1S_1185 is a predicted M23/M37 family metalloendopeptidase, which is a class of enzymes involved in cleaving peptidoglycan for cell growth and division. A1S_0408 is a putative glutathione *S*-transferase, which is a family of enzymes that contribute to detoxification of endogenous or xenobiotic compounds and oxidative stress metabolism to repair damaged cellular components (27). With such broad functions, more research is needed on these proteins to understand how they specifically contribute to OXA-23 overexpression tolerance and PG homeostasis in A. baumannii.

To follow up on the increased amidase activity associated with OXA-23 overexpression in our PG composition studies, we attempted to demonstrate that purified OXA-23 enzyme directly cleaves purified PG sacculi, but we were unable to confirm OXA-23 amidase activity *in vitro* using a variety of conditions (see Fig. S2 in the supplemental material). Therefore, it is possible that OXA-23 localizes with and/or activates another enzyme that performs the amidase activity. In fact, an *in vivo* cross-linking study in A. baumannii AB5075 revealed that OXA-23 interacts with 18 periplasmic proteins directly and another 4 proteins by extended interaction partners. These interaction partners represent several different functional classes, including porins (i.e., OmpA, OmpW, CarO), virulence factors (i.e., EcnB, hemolysins), metabolic enzymes (i.e., SodC), and uncharacterized proteins (28, 29). It is possible that under normal conditions, all OXA-23 enzymes would be interacting with these known protein partners; however, OXA-23 overexpression may provide additional binding opportunities for other unidentified periplasmic proteins, directly or in an extended complex, that may drive the collateral physiological damage we have shown here.

The interplay of antibiotic resistance, virulence, and peptidoglycan biology remains a controversial and complex topic (30, 31). Our study has shed some light on this relationship in A. baumannii as well as revealed novel MDR-specific antimicrobial targets that may limit off-target effects on the host microbiome. However, there are many more questions left to be addressed. For example, what are the collateral effects of overexpressing other β-lactamases? There have been over 1,000 OXA variants reported so far in addition to the other classes of β-lactamases, each with their own unique spectrum of activities, which may impact cellular physiology differently. We are also interested in how these effects are impacted by the presence of other antibiotic resistance mechanisms. In our study, we focused on the direct influence of OXA-23 overexpression in an antibiotic-sensitive strain. However, MDR A. baumannii is defined by the presence of at least three antibiotic resistance mechanisms, and to our knowledge, no publications break down the individual physiological impact of several resistance genes in a single pathogen.

In conclusion, the overexpression of OXA-23 in A. baumannii is associated with PG composition changes without an apparent fitness cost. However, the collateral physiological damage must be compensated for, such that genes that were dispensable for wild-type growth became conditionally essential. Furthermore, the consistent connections we observed between OXA-23 overexpression and PG biosynthesis- and homeostasis-related enzymes support the hypothesis that β-lactamases may still maintain residual PBP function (32). Most importantly, OXA class β-lactamases are widely disseminated among Gram-negative *Enterobacteriaceae* suggesting that our results may reveal broad-spectrum antimicrobial targets that simultaneously target β-lactam resistance and cell viability in addition to identifying putative PG-related activities for OXA-23 β-lactamase in other MDR pathogens, such as E. coli, P. aeruginosa, and Klebsiella pneumoniae. This work paves the way for the identification of new antimicrobial targets, where inhibitors would selectively kill β-lactamase-expressing strains.

## MATERIALS AND METHODS

### Bacterial strains, growth conditions, and antibiotics.

Bacterial strains and plasmids used in this study are listed in Tables S3 and S4 in the supplemental material. Primer sequences are available upon request. Acinetobacter baumannii ATCC 17978, AB5075, and AB5075 Δ*wzc* were from lab stocks. Escherichia coli EC100D was used for plasmid propagation (Epicentre, Madison, WI). A. baumannii and E. coli were cultured in Luria-Bertani broth (LB). Where indicated, medium was supplemented with kanamycin (KAN; 30 μg/ml), tetracycline (TET; 5 μg/ml), apramycin (APR; 40 μg/ml), or hygromycin (HYG; 150 μg/ml) for plasmid maintenance or transposon selection (Sigma-Aldrich, St. Louis, MO).

10.1128/mBio.03137-21.3 TABLE S3 Bacterial strains used in this study. Download Table S3, DOCX file, 0.02 MB. Copyright © 2021 Colquhoun et al. 2021 Colquhoun et al. https://creativecommons.org/licenses/by/4.0/ This content is distributed under the terms of the Creative Commons Attribution 4.0 International license.

10.1128/mBio.03137-21.4 TABLE S4 Plasmids used in this study. Download Table S4, DOCX file, 0.02 MB. Copyright © 2021 Colquhoun et al. 2021 Colquhoun et al. https://creativecommons.org/licenses/by/4.0/ This content is distributed under the terms of the Creative Commons Attribution 4.0 International license.

### Electroporation of ATCC 17978.

Electroporations were conducted with cultures of strain ATCC 17978 grown at 37°C with shaking to late log phase. Cells were pelleted by centrifugation and washed three times with molecular-grade water. Cells were mixed with either genomic DNA (gDNA) or plasmid DNA in 0.2-cm cuvettes and electroporated at 2.50 kV. Transformants were recovered in 1 ml LB for 30 min at 37°C without shaking followed by 1 h at 37°C with shaking. Transformants were then selected for by plating on the appropriate antibiotic.

### Construction of an A. baumannii 17978 strain with an inducible OXA-23 gene.

A mini-Tn*7* transposon system was used to generate an IPTG-inducible copy of the *bla*_OXA-23_ gene in single copy. To construct the mini-Tn*7*-containing ptac-controlled expression plasmid, pTn7::*bla*_OXA-23_, a DNA fragment beginning 30 bp upstream from the predicted start codon and ending 20 bp downstream from the predicted stop codon, was amplified by PCR using chromosomal DNA from A. baumannii strain AB5075 as the template (Phusion Hot Start Polymerase; Thermo Fisher Scientific, Waltham, MA). The fragment was purified from an agarose gel slice and ligated into pUC18-mini-Tn7-LAC-Apra (33) that had been digested with SmaI (New England Biolabs, Ipswich, MA) using a Fast-Link ligation kit (Epicentre). The ligation was transformed into E. coli TransforMax EC100D competent cells and selected on LB agar with apramycin. Plasmids containing the β-lactamase gene insertion were identified by size shift and verified by sequencing.

To incorporate the IPTG-inducible *bla*_OXA-23_ gene into the *attTn7* locus within the ATCC 17978 chromosome, electrocompetent ATCC 17978 was coelectroporated with pTn7::*bla*_OXA-23_ and pTNS2, and transformants were selected on LB agar with apramycin, resulting in the strain ATCC 17978 Tn*7*::*bla*_OXA-23_. Successful mini-Tn*7* integrants were confirmed by PCR using primers outside the Tn*7* insertion site near *glmS*.

A catalytically inactive OXA-23 variant (S79A) was generated using a QuikChange II XL site-directed mutagenesis kit (Agilent Technologies, Santa Clara, CA). Briefly, pTn7::*bla*_OXA-23_ was amplified using mutagenesis primers according to the manufacturer’s instructions. The parental plasmid was digested with DpnI, and the remaining mutagenized plasmid was transformed into TransforMax EC100D electrocompetent E. coli. Transformants were selected for using apramycin, and the mutation was confirmed by sequencing. The mutagenized mini-Tn*7*-containing plasmid was then electroporated together with pTNS2 into strain ATCC 17978 and confirmed as described above.

A plasmid constitutively expressing the OXA-23 β-lactamase, pQF1266-hyg::*bla*_OXA-23_, was generated by cloning the *bla*_OXA-23_ DNA fragment into pQF1266-hyg digested with SmaI. The ligation was transformed into E. coli TransforMax EC100D competent cells and selected for on LB agar with Hyg containing 5-bromo-4-chloro-3-indolyl-β-d-galactopyranoside (X-Gal; Sigma-Aldrich). White colonies indicated insert integration, and fragment orientation was confirmed by sequencing.

### Total bacterial RNA purification.

Total A. baumannii RNA was purified as previously described (34). Briefly, cultures of A. baumannii ATCC 17978 control and ATCC 17978 Tn*7*::*bla*_OXA-23_ were grown in LB with or without 2 mM isopropyl-β-d-1-thiogalactopyranoside (IPTG; Sigma-Aldrich) at 37°C with shaking until they reached an optical density at 600 nm (OD_600_) of ∼0.8. The cells were harvested from cultures by centrifugation, and RNA was isolated using a MasterPure RNA purification kit (Epicentre) according to the manufacturer’s protocol. Contaminating DNA was removed by treatment using a TURBO DNA-free kit (Ambion, Austin, TX) according to the manufacturer’s protocol. The RNA concentration was quantified using a NanoDrop ND-1000 spectrophotometer (Thermo Fisher Scientific). To confirm that samples were not contaminated with DNA following DNase treatment, PCR of RNA samples was performed.

### RNA-Seq and analysis.

RNA-Seq was performed in triplicate using samples generated from three independent cultures of ATCC 17978 control and ATCC 17978 Tn*7*::*bla*_OXA-23_ grown with 2 mM IPTG to an OD_600_ of ∼0.8. Cell pellets were sent to Genewiz for processing, library preparation, and RNA sequencing. Paired-end Illumina libraries were mapped against the A. baumannii ATCC 17978-mmf genome (CP012004.1) using Bowtie2 aligner (version 2.4.2), and differential gene expression was quantified by DESeq2 (version 2.11.40.6; University of Maryland Genomics Resource Center). Fisher’s exact test (modified by DESeq2) was used to calculate the *P* values, which were adjusted for multiple testing by the Benjamini-Hochberg method. Differentially expressed transcripts with a *P* value of ≤0.05, a false discovery rate of ≤0.05, and a log_2_ fold change of ≥1.5 were used. RNA-Seq data sets have been deposited to the NCBI’s Gene Expression Omnibus and are accessible through GSE185203 with individual files under GSM5608026, GSM5608027, GSM5608028, GSM5608029, GSM5608030, and GSM5608031.

### Quantitative reverse transcription-PCR (qRT-PCR).

Total RNA (1 μg) was used to prepare cDNA using the iScript cDNA synthesis kit (Bio-Rad) with random primers as described by the manufacturer. Reaction mixtures lacking reverse transcriptase served as a control for the presence of contaminating DNA. cDNA reactions and controls were then diluted 1:10 with sterile water and used as a template for qRT-PCR. Data were generated using cDNA prepared from three independent RNA isolations, and qRT-PCRs were performed in technical triplicates to ensure accuracy. Fold changes in gene expression relative to the control strain (ATCC 17978) and a control gene (16S rRNA gene) were determined using the 2^−ΔΔ^*^CT^* method (35).

### Peptidoglycan isolation.

Peptidoglycan sacculi were isolated from cultures of A. baumannii ATCC 17978 control and ATCC 17978 Tn7::*bla*_OXA-23_ grown in 200 ml LB with or without 2 mM IPTG at 37°C with shaking to an OD_600_ of ∼0.8 as described previously (36). Briefly, frozen cell pellets were suspended in 20 ml of cold (4°C) 20 mM sodium phosphate buffer, pH 7.5 (Sigma). The suspension was added dropwise to an equal volume of boiling 8% sodium dodecyl sulfate (SDS) (Sigma) in 20 mM sodium phosphate buffer, pH 7.5. The samples were boiled with stirring for 3 h and allowed to cool overnight. The SDS was removed by repeated ultracentrifugation at 45,000 × *g* for 30 min and washing in 20 mM sodium phosphate buffer, pH 7.5, four times. Washed sacculi were suspended in 1 ml of 20 mM sodium phosphate buffer, pH 6.0, and incubated with 100 μg/ml amylase (Sigma), 10 μg/ml DNase I (Sigma), 50 μg/ml RNase A (Sigma), and 20 mM MgCl_2_ (Sigma) for 1 h at 37°C. Next, 200 μg/ml pronase (Roche, Basel, Switzerland) was added and incubated for an additional 18 h at 37°C with gentle rocking. Samples were then boiled in 4% SDS for 3 h with stirring and allowed to cool overnight. The removal of SDS by repeated ultracentrifugation at 45,000 × *g* for 30 min and washing with 20 mM sodium phosphate buffer, pH 7.5, four times. Washed sacculi were suspended in 1 ml of 20 mM sodium phosphate buffer, pH 7.5, and lyophilized. The lyophilized PG sacculi were weighed and stored at −20°C until prepared for quantification and mass spectrometry.

### Peptidoglycan compositional analysis.

Lyophilized peptidoglycan sacculi were suspended to 10 mg/ml in H_2_O and sonicated for 20 s at 30% amplitude to disperse clumps. To produce muropeptides, the suspended sacculi were digested with 100 μg/ml mutanolysin in 100 mM ammonium acetate (pH 5.5) and 50 mM MgCl_2_ for 18 h at 37°C. Soluble muropeptides in the mutanolysin digests were separated by reverse-phase high-pressure liquid chromatography (RP-HPLC) on a 4.6 mm × 250 mm Gemini C_18_ (5-μm) analytical column (Phenomenex Inc., Torrance, CA). Samples were applied to the column, previously equilibrated in 50 mM sodium phosphate buffer, pH 4.3, at a flow rate of 0.5 ml min^−1^. After buffer salts eluted in the void volume (10 min), a linear gradient to 15% methanol in 50 mM sodium phosphate buffer (pH 5.1) was applied over 120 min. Detection was achieved by monitoring absorbance at 205 nm. Fractions of interest were collected by hand for subsequent liquid chromatograph-mass spectrometric (LC-MS) analyses.

LC-MS analyses were performed on an Agilent 1200 HPLC liquid chromatograph interfaced with an Agilent UHD 6540 Q-TOF mass spectrometer at the Mass Spectrometry Facility of the Advanced Analysis Centre, University of Guelph as described previously (17). The mass spectrometer electrospray capillary voltage was maintained at 4.0 kV, and the drying gas temperature was set at 350°C with a flow rate of 13 liters min^−1^. Nebulizer pressure was 40 lb/in^2^, and the fragmentor was set to 150 V. Nitrogen was used as both nebulizing and drying gas and collision-induced gas. The mass-to-charge (*m/z*) ratio was scanned across the *m/z* range of 200 to 2,000 *m/z* in 4 GHz extended dynamic range positive-ion auto tandem mass spectrometry (MS/MS) mode. Three precursor ions per cycle were selected for fragmentation. The instrument was externally calibrated with the electrospray ionization (ESI) TuneMix (Agilent Technologies Inc.). The sample injection volume was 10 μl. Triplicate technical replicates were performed for all biological replicates.

### OXA-23 protein purification.

OXA-23 β-lactamase was purified as previously described (37). E. coli BL21(DE3) containing a pET28(+)::*bla*_OXA-23_ construct were grown in super optimal broth (SOB) containing 50 μg/ml kanamycin at 37°C in shaker flasks to achieve an OD_600_ of 0.8. IPTG (isopropyl-β-d-1-thiogalactopyranoside) was added to the culture to a final concentration of 0.2 mM, and the culture was grown for three more hours. The cells were centrifuged and frozen overnight at −20°C. Cells were lysed using a QIAexpress nickel-nitrilotriacetic acid (Ni-NTA) fast-start kit, followed by nickel column purification of the His-tagged protein according to the manufacturer’s protocol (Qiagen Inc., Valencia, CA). To remove the His tag, the eluted protein was incubated with thrombin (Novagen, Madison, WI) overnight at 4°C (1.6 U per mg protein). The cleaved protein was separated from the 6×His tag peptides by size exclusion chromatography using a HiLoad 16/60 Superdex 75 column (GE Healthcare Life Science).

### *In vitro* nitrocefin cleavage assay.

β-Lactamase activity of purified OXA-23 was confirmed using a nitrocefin assay kit (MAK221; Sigma). Briefly, dilutions of purified OXA-23 in β-lactamase hydrolysis buffer were combined with 2 μl nitrocefin in a 100-μl reaction mixture. Cleavage of nitrocefin was monitored by measuring the absorbance at 490 nm every minute for 1 h at room temperature.

### *In vitro* peptidoglycan amidase activity assay.

Amidase activity of purified OXA-23 was determined as described previously (38). Briefly, isolated A. baumannii peptidoglycan was incubated in 20 mM Remazol Brilliant Blue R (RBB; Sigma) in 0.25 M NaOH with gentle rocking overnight at 37°C. The next day, the solution was neutralized with HCl and RBB-stained peptidoglycan repeatedly washed with water and pelleted by centrifugation until the supernatant was clear. The final pellet was resuspended in water to 100 mg/ml and stored at −20°C. For peptidoglycan cleavage determination, 1 mg of RBB-stained A. baumannii sacculi was incubated with 0 or 20 μM OXA-23 or 4 μM lysozyme (positive control) in 100 μl phosphate-buffered saline (PBS) (10 mM Na_2_HPO_4_, 2 mM KH_2_PO_4_, 137 mM NaCl, and 2.7 mM KCl [pH 7.4]) at 37°C for 24 h. Reactions were stopped by incubating at 95°C for 5 min. Following termination, all reactions were centrifuged at 10,000 × *g* for 1 min at room temperature. Supernatants were transferred to a 96-well flat-bottom plate, and the absorbance at 595 nm was measured.

For cofactor supplementation assays, MgCl_2_, ZnCl_2_, MnCl_2_, NiCl_2_, CoCl_2_, CaCl_2_, or FeCl_2_ was added to a final concentration of 4 mM. For pH titration assays, 10× PBS solution was adjusted to pH 5, 6, 7, 8, or 9 using NaOH or HCl and then diluted to 1× in the final reaction. For whole-cell lysate add-back assays, an avirulent A. baumannii strain (*Δwzc*) was grown shaking to an OD_600_ of 0.8 and pelleted by centrifugation. The cell pellet was resuspended in ice-cold buffer C (50 mM Na_2_HPO_4_, 150 mM NaCl [pH 7.0]) and lysed by passing through the French press three times at 16,000 × *g*. Cell debris was pelleted by centrifugation at 14,000 × *g* for 15 min at 4°C. The supernatant was collected, passed through a 0.22-μm filter, and stored at 4°C. Increasing volumes of this lysate were added to the 20 μM OXA-23 reaction mixtures detailed above.

### MIC.

MICs were determined using a modified Etest assay as previously described (39). Briefly, two strains at an OD_600_ of 0.1 were inoculated onto either side of an Etest strip (bioMérieux, Marcy-l'Étoile, France) by pipetting 10 μl of culture next to the bottom of the strip, tilting the plate to spread the culture up the side of the strip, and pipetting away excess culture at the top of the strip. MICs were assessed after 16-h incubation at 37°C on LB agar plates with and without 5 mM IPTG. Experiments were performed two independent times to confirm the reproducibility of trends.

### Random transposon library generation and screening for conditionally essential genes.

To create an insertional library, A. baumannii ATCC 17978 Tn*7*::*bla*_OXA-23_ was mutagenized using the EZ-Tn5 <Kan-2> transposon kit (Lucigen, Middleton, WI) according to the manufacturer’s instructions. Briefly, ATCC 17978 Tn*7*::*bla*_OXA-23_ was grown to an OD_600_ of 0.8 in 2 ml LB and pelleted by centrifugation. Cells were washed three times and resuspended in 120 μl nuclease-free water. Cells (70 μl) were electroporated with 1 μl EZ-Tn5 <Kan-2> Tnp, plated on LB agar with kanamycin, and incubated overnight at 37°C. Approximately 100,000 resulting individual colonies were pooled and used for screening experiments.

To screen for insertions that become conditionally essential upon OXA-23 overexpression, individual A. baumannii colonies containing random transposon insertions were patched onto LB agar containing 30 μg/ml KAN and LB agar containing 30 μg/ml KAN and 5 mM IPTG and incubated at 37°C overnight. Patches that failed to grow on IPTG were cultured, and genomic DNA was isolated as described below. Genomic DNAs from mutants were subjected to a partial Sau3AI digestion. Fragments in the 2- to 5-kb range were gel purified, ligated to pACYC184 (40), and digested with BamHI. The resulting ligation product was electroporated into EC100D competent cells and plated onto LB agar plates containing chloramphenicol (25 μg/ml) and kanamycin (20 μg/ml). Plasmids were sequenced with the FP1 and RP1 primers, which read outward from the transposon, and the chromosomal region disrupted by the transposon was determined by BLAST analysis.

### Genomic DNA isolation.

Cultures used for genomic DNA (gDNA) preparations were grown with shaking at 37°C overnight in LB with the appropriate antibiotics. One milliliter of cells was pelleted through centrifugation and then resuspended in Tris-EDTA. Cells were lysed by incubation for 1 h at 37°C with 0.5% SDS and 400 μg/ml proteinase K. Sodium chloride was added to a final concentration of 0.7 M, and DNA was extracted twice using equal volumes of phenol-chloroform and isoamyl alcohol. DNA was isolated by mixing with 2.5 volumes of 95% ethanol until a precipitate was visible. DNA pellets were collected by centrifugation, washed twice with 75% ethanol, dried, and resuspended in molecular-grade water.

### Complementation analysis.

The wild-type *A1S_1185* and *A1S_0408* genes were amplified by PCR using the primers listed in Table S2. Each PCR product contained its native ribosome binding site and was cloned into the SmaI site of pQF1266-hyg for expression from an endogenous promoter in the plasmid. The ligation was transformed into E. coli TransforMax EC100D competent cells and selected on LB agar with hygromycin and X-Gal. White colonies indicated insert integration, and fragment orientation was confirmed by sequencing.

### Characterization of mutants that become conditionally essential during OXA-23 β-lactamase overexpression.

Genomic DNA from strains with transposon insertions in genes that became essential upon β-lactamase overexpression was freshly electroporated into ATCC 17978 Tn*7*::*bla*_OXA-23_, selected on LB plus KAN agar, and incubated for 16 h at 28°C. Well-isolated transformants were picked and resuspended in 50 μl LB. Resuspensions were serially diluted and spotted onto LB plus KAN agar with and without 5 mM IPTG and incubated overnight at 37°C. For electroporations with *A1S_1185*::*kan* gDNA, results were highly variable, and a closely linked suppressor mutation was considered as being responsible for the variability. Therefore, recombineering with pAT04 was used as described previously (21, 41) to recreate the mutation using a PCR fragment that contained only the *A1S_1185* coding region with the disruption. Dilutions of transformant colonies were then plated on KAN plates with and without IPTG to determine viability.

To assess IPTG sensitivity of conditional essential mutants in wild-type and catalytically inactive OXA-23, conditional essential mutant gDNA was freshly electroporated into A. baumannii ATCC 17978 Tn*7*::*bla*_OXA-23_ and ATCC 17978 Tn*7*:: *bla*_OXA-23_ (S79A) strains, 250 μl recovery plated on LB plus KAN agar with and without 5 mM IPTG, and incubated for 24 h at 37°C. CFU were enumerated from each plate, and the percent IPTG sensitivity calculated by dividing the number of CFU on the IPTG plate by the number of CFU on the control plate (CFU_+IPTG_/CFU_Control_ × 100).

To evaluate complementation of the IPTG sensitivity phenotype, the same process as described above was followed. Conditional essential mutant gDNA was freshly electroporated into ATCC 17978 Tn*7*::*bla*_OXA-23_ pQF1266-hyg vector or pWH1266 vector and ATCC 17978 Tn*7*::*bla*_OXA-23_ with the appropriate pQF1266-hyg or pWH1266 complementation plasmid, 250 μl recovery plated on LB plus KAN plus HYG or TET agar with and without 5 mM IPTG and incubated for 24 h at 37°C, and the percent IPTG sensitivity was calculated.

### Bacterial growth curves.

Genomic DNA from strains with transposon insertions in genes that became essential upon β-lactamase overexpression was freshly electroporated into strain ATCC 17978 Tn*7*::*bla*_OXA-23_, selected on LB plus Kan agar, and incubated for 16 h at 28°C. Well-isolated transformants were picked and used to inoculate cultures that were grown overnight without shaking at 28°C in LB with Kan. Cultures were normalized to an OD_600_ of 0.05 in LB with or with 5 mM IPTG and grown at 37°C with shaking with OD_600_ measurements every 30 min for 8 h.

### Phase-contrast microscopy.

ATCC 17978 Tn7::*bla*_OXA-23_ containing the conditionally essential mutants was grown to mid-log phase (OD_600_ of 0.4) in LB broth with or without 5 mM IPTG, pelleted, and resuspended in a 1/10 volume of LB broth. Images were taken by phase-contrast microscopy using an Olympus BX51 microscope (Waltham, MA) and photographed with an Infinity 2-1 charge-coupled-device (CCD) camera (Lumenera, Ottawa, Canada). The magnification was ×1,000.

### Generation of CRISPRi-mediated *murA* knockdown strain.

A plasmid encoding a *murA*-directed single guide RNA (sgRNA) for targeted CRISPRi was constructed by PCR amplifying the gRNA scaffold region of pJE53 as described in reference 42. Briefly, using a forward primer containing a 24-base targeting sequence and SpeI site at its 5′ end, a reverse primer with ApaI in its 5′ end (Table S2), and Phusion Hot Start Polymerase, the PCR product was digested with SpeI and ApaI, cloned by replacing the SpeI-ApaI guide fragment in pJE53, and confirmed by sequencing. pJE53::murA-sgRNA was introduced into YDA004 via electroporation.

### CRISPRi knockdown or chemical inhibition and cell survival.

To evaluate the synergistic lethality of a *murA* knockdown combined with OXA-23 overexpression, ATCC 17978 Tn*7*::dCas9 pJE53::murA-sgRNA was electroporated with pQF1266-hyg vector and pQF1266-hyg::*bla*_OXA-23_ and selected for on LB plus Kan plus Hyg. Well-isolated transformants were picked and resuspended in 50 μl LB. Resuspensions were seriallydiluted and spotted onto LB plus KAN plus HYG agar with increasing concentrations of anhydrotetracycline (ATc; Sigma-Aldrich) and incubated overnight at 37°C.

To evaluate the synergistic lethality of peptidoglycan synthesis chemical inhibition combined with OXA-23 overexpression, ATCC 17978 Tn*7*::dCas9 was electroporated with pQF1266-hyg vector and pQF1266-hyg::*bla*_OXA-23_ and selected for on LB plus Hyg. Well-isolated transformants were picked and resuspended in 50 μl LB. Resuspensions were serially diluted and spotted onto LB plus HYG agar with increasing concentrations of fosfomycin (FOF; Sigma-Aldrich), d-cycloserine (Dc; Sigma-Aldrich), or ciprofloxacin (CIP; Sigma-Aldrich) and incubated overnight at 37°C.

### Data availability.

RNA-Seq data sets have been deposited to the NCBI’s Gene Expression Omnibus and are accessible through GSE185203 with individual files under GSM5608026, GSM5608027, GSM5608028, GSM5608029, GSM5608030, and GSM5608031.

## References

[1] World Health Organization. 2014. Antimicrobial resistance: global report on surveillance 2014. World Health Organization, Geneva, Switzerland.

[2] Merkier AK, Centron D. 2006. bla(OXA-51)-type beta-lactamase genes are ubiquitous and vary within a strain in Acinetobacter baumannii. Int J Antimicrob Agents 28:110–113. doi:10.1016/j.ijantimicag.2006.03.023. 16844350

[3] Heritier C, Poirel L, Fournier PE, Claverie JM, Raoult D, Nordmann P. 2005. Characterization of the naturally occurring oxacillinase of Acinetobacter baumannii. Antimicrob Agents Chemother 49:4174–4179. doi:10.1128/AAC.49.10.4174-4179.2005. 16189095 PMC1251506

[4] Hall BG, Barlow M. 2004. Evolution of the serine beta-lactamases: past, present and future. Drug Resist Updat 7:111–123. doi:10.1016/j.drup.2004.02.003. 15158767

[5] Holtje JV. 1998. Growth of the stress-bearing and shape-maintaining murein sacculus of Escherichia coli. Microbiol Mol Biol Rev 62:181–203. doi:10.1128/MMBR.62.1.181-203.1998. 9529891 PMC98910

[6] Nanninga N. 1998. Morphogenesis of Escherichia coli. Microbiol Mol Biol Rev 62:110–129. doi:10.1128/MMBR.62.1.110-129.1998. 9529889 PMC98908

[7] Chang YH, Labgold MR, Richards JH. 1990. Altering enzymatic activity: recruitment of carboxypeptidase activity into an RTEM beta-lactamase/penicillin-binding protein 5 chimera. Proc Natl Acad Sci USA 87:2823–2827. doi:10.1073/pnas.87.7.2823. 2181451 PMC53783

[8] Rhazi N, Galleni M, Page MI, Frere JM. 1999. Peptidase activity of beta-lactamases. Biochem J 341:409–413. doi:10.1042/bj3410409. 10393100 PMC1220374

[9] Sun T, Nukaga M, Mayama K, Braswell EH, Knox JR. 2003. Comparison of beta-lactamases of classes A and D: 1.5-A crystallographic structure of the class D OXA-1 oxacillinase. Protein Sci 12:82–91. doi:10.1110/ps.0224303. 12493831 PMC2312410

[10] Fernandez A, Perez A, Ayala JA, Mallo S, Rumbo-Feal S, Tomas M, Poza M, Bou G. 2012. Expression of OXA-type and SFO-1 beta-lactamases induces changes in peptidoglycan composition and affects bacterial fitness. Antimicrob Agents Chemother 56:1877–1884. doi:10.1128/AAC.05402-11. 22290977 PMC3318342

[11] Gallant CV, Daniels C, Leung JM, Ghosh AS, Young KD, Kotra LP, Burrows LL. 2005. Common beta-lactamases inhibit bacterial biofilm formation. Mol Microbiol 58:1012–1024. doi:10.1111/j.1365-2958.2005.04892.x. 16262787 PMC3097517

[12] Morosini MI, Ayala JA, Baquero F, Martinez JL, Blazquez J. 2000. Biological cost of AmpC production for Salmonella enterica serotype Typhimurium. Antimicrob Agents Chemother 44:3137–3143. doi:10.1128/AAC.44.11.3137-3143.2000. 11036037 PMC101617

[13] Perez-Gallego M, Torrens G, Castillo-Vera J, Moya B, Zamorano L, Cabot G, Hultenby K, Alberti S, Mellroth P, Henriques-Normark B, Normark S, Oliver A, Juan C. 2016. Impact of AmpC derepression on fitness and virulence: the mechanism or the pathway? mBio 7:e01783-16. doi:10.1128/mBio.01783-16. 27795406 PMC5080387

[14] Torrens G, Perez-Gallego M, Moya B, Munar-Bestard M, Zamorano L, Cabot G, Blazquez J, Ayala JA, Oliver A, Juan C. 2017. Targeting the permeability barrier and peptidoglycan recycling pathways to disarm Pseudomonas aeruginosa against the innate immune system. PLoS One 12:e0181932. doi:10.1371/journal.pone.0181932. 28742861 PMC5526577

[15] Smith CA, Antunes NT, Stewart NK, Toth M, Kumarasiri M, Chang M, Mobashery S, Vakulenko SB. 2013. Structural basis for carbapenemase activity of the OXA-23 beta-lactamase from Acinetobacter baumannii. Chem Biol 20:1107–1115. doi:10.1016/j.chembiol.2013.07.015. 24012371 PMC3888872

[16] Mugnier PD, Poirel L, Naas T, Nordmann P. 2010. Worldwide dissemination of the blaOXA-23 carbapenemase gene of Acinetobacter baumannii. Emerg Infect Dis 16:35–40. doi:10.3201/eid1601.090852. 20031040 PMC2874364

[17] Hamidian M, Nigro SJ. 2019. Emergence, molecular mechanisms and global spread of carbapenem-resistant Acinetobacter baumannii. Microb Genom 5:e000306. doi:10.1099/mgen.0.000306. PMC6861865 31599224

[18] Vollmer W, Joris B, Charlier P, Foster S. 2008. Bacterial peptidoglycan (murein) hydrolases. FEMS Microbiol Rev 32:259–286. doi:10.1111/j.1574-6976.2007.00099.x. 18266855

[19] Vermassen A, Leroy S, Talon R, Provot C, Popowska M, Desvaux M. 2019. Cell wall hydrolases in bacteria: insight on the diversity of cell wall amidases, glycosidases and peptidases toward peptidoglycan. Front Microbiol 10:331. doi:10.3389/fmicb.2019.00331. 30873139 PMC6403190

[20] Zhou R, Chen S, Recsei P. 1988. A dye release assay for determination of lysostaphin activity. Anal Biochem 171:141–144. doi:10.1016/0003-2697(88)90134-0. 3407910

[21] Knight D, Dimitrova DD, Rudin SD, Bonomo RA, Rather PN. 2016. Mutations decreasing intrinsic beta-lactam resistance are linked to cell division in the nosocomial pathogen Acinetobacter baumannii. Antimicrob Agents Chemother 60:3751–3758. doi:10.1128/AAC.00361-16. 27067318 PMC4879375

[22] Marquardt JL, Siegele DA, Kolter R, Walsh CT. 1992. Cloning and sequencing of Escherichia coli murZ and purification of its product, a UDP-N-acetylglucosamine enolpyruvyl transferase. J Bacteriol 174:5748–5752. doi:10.1128/jb.174.17.5748-5752.1992. 1512209 PMC206525

[23] Neuhaus FC, Lynch JL. 1964. The enzymatic synthesis of D-alanyl-D-alanine. III. On the inhibition of D-alanyl-D-alanine synthetase by the antibiotic D-cycloserine. Biochemistry 3:471–480. doi:10.1021/bi00892a001. 14188160

[24] Cordeiro NF, Chabalgoity JA, Yim L, Vignoli R. 2014. Synthesis of metallo-beta-lactamase VIM-2 is associated with a fitness reduction in Salmonella enterica serovar Typhimurium. Antimicrob Agents Chemother 58:6528–6535. doi:10.1128/AAC.02847-14. 25136026 PMC4249381

[25] Pazos M, Natale P, Vicente M. 2013. A specific role for the ZipA protein in cell division: stabilization of the FtsZ protein. J Biol Chem 288:3219–3226. doi:10.1074/jbc.M112.434944. 23233671 PMC3561543

[26] Pazos M, Peters K, Casanova M, Palacios P, VanNieuwenhze M, Breukink E, Vicente M, Vollmer W. 2018. Z-ring membrane anchors associate with cell wall synthases to initiate bacterial cell division. Nat Commun 9:5090. doi:10.1038/s41467-018-07559-2. 30504892 PMC6269477

[27] Vuilleumier S. 1997. Bacterial glutathione S-transferases: what are they good for? J Bacteriol 179:1431–1441. doi:10.1128/jb.179.5.1431-1441.1997. 9045797 PMC178850

[28] Wu X, Chavez JD, Schweppe DK, Zheng C, Weisbrod CR, Eng JK, Murali A, Lee SA, Ramage E, Gallagher LA, Kulasekara HD, Edrozo ME, Kamischke CN, Brittnacher MJ, Miller SI, Singh PK, Manoil C, Bruce JE. 2016. In vivo protein interaction network analysis reveals porin-localized antibiotic inactivation in Acinetobacter baumannii strain AB5075. Nat Commun 7:13414. doi:10.1038/ncomms13414. 27834373 PMC5114622

[29] Zhong X, Wu X, Schweppe DK, Chavez JD, Mathay M, Eng JK, Keller A, Bruce JE. 2020. In vivo cross-linking MS reveals conservation in OmpA linkage to different classes of beta-lactamase enzymes. J Am Soc Mass Spectrom 31:190–195. doi:10.1021/jasms.9b00021. 32031408 PMC7970438

[30] Beceiro A, Tomas M, Bou G. 2013. Antimicrobial resistance and virulence: a successful or deleterious association in the bacterial world? Clin Microbiol Rev 26:185–230. doi:10.1128/CMR.00059-12. 23554414 PMC3623377

[31] Juan C, Torrens G, Barcelo IM, Oliver A. 2018. Interplay between peptidoglycan biology and virulence in Gram-negative pathogens. Microbiol Mol Biol Rev 82:e00033-18. doi:10.1128/MMBR.00033-18. 30209071 PMC6298613

[32] Massova I, Mobashery S. 1998. Kinship and diversification of bacterial penicillin-binding proteins and beta-lactamases. Antimicrob Agents Chemother 42:1–17. doi:10.1128/AAC.42.1.1. 9449253 PMC105448

[33] Choi KH, Gaynor JB, White KG, Lopez C, Bosio CM, Karkhoff-Schweizer RR, Schweizer HP. 2005. A Tn7-based broad-range bacterial cloning and expression system. Nat Methods 2:443–448. doi:10.1038/nmeth765. 15908923

[34] Chin CY, Tipton KA, Farokhyfar M, Burd EM, Weiss DS, Rather PN. 2018. A high-frequency phenotypic switch links bacterial virulence and environmental survival in Acinetobacter baumannii. Nat Microbiol 3:563–569. doi:10.1038/s41564-018-0151-5. 29693659 PMC5921939

[35] Schmittgen TD, Livak KJ. 2008. Analyzing real-time PCR data by the comparative C(T) method. Nat Protoc 3:1101–1108. doi:10.1038/nprot.2008.73. 18546601

[36] Schaub RE, Dillard JP. 2017. Digestion of peptidoglycan and analysis of soluble fragments. Bio Protoc 7:e2438. doi:10.21769/BioProtoc.2438. PMC5602577 28932761

[37] Papp-Wallace KM, Nguyen NQ, Jacobs MR, Bethel CR, Barnes MD, Kumar V, Bajaksouzian S, Rudin SD, Rather PN, Bhavsar S, Ravikumar T, Deshpande PK, Patil V, Yeole R, Bhagwat SS, Patel MV, van den Akker F, Bonomo RA. 2018. Strategic approaches to overcome resistance against Gram-negative pathogens using beta-lactamase inhibitors and beta-lactam enhancers: activity of three novel diazabicyclooctanes WCK 5153, zidebactam (WCK 5107), and WCK 4234. J Med Chem 61:4067–4086. doi:10.1021/acs.jmedchem.8b00091. 29627985 PMC6131718

[38] Uehara T, Parzych KR, Dinh T, Bernhardt TG. 2010. Daughter cell separation is controlled by cytokinetic ring-activated cell wall hydrolysis. EMBO J 29:1412–1422. doi:10.1038/emboj.2010.36. 20300061 PMC2868575

[39] Anderson SE, Sherman EX, Weiss DS, Rather PN. 2018. Aminoglycoside heteroresistance in Acinetobacter baumannii AB5075. mSphere 3:e00271-18. doi:10.1128/mSphere.00271-18. 30111627 PMC6094062

[40] Chang AC, Cohen SN. 1978. Construction and characterization of amplifiable multicopy DNA cloning vehicles derived from the P15A cryptic miniplasmid. J Bacteriol 134:1141–1156. doi:10.1128/jb.134.3.1141-1156.1978. 149110 PMC222365

[41] Tucker AT, Nowicki EM, Boll JM, Knauf GA, Burdis NC, Trent MS, Davies BW. 2014. Defining gene-phenotype relationships in Acinetobacter baumannii through one-step chromosomal gene inactivation. mBio 5:e01313-14. doi:10.1128/mBio.01313-14. 25096877 PMC4128354

[42] Bai J, Dai Y, Farinha A, Tang AY, Syal S, Vargas-Cuebas G, van Opijnen T, Isberg RR, Geisinger E. 2021. Essential gene analysis in Acinetobacter baumannii by high-density transposon mutagenesis and CRISPR interference. J Bacteriol 203:e00565-20. doi:10.1128/JB.00565-20. PMC8316057 33782056

