# Supplementary figures and images for: OXA-23 β-Lactamase Overexpression in Acinetobacter baumannii Drives Physiological Changes Resulting in New Genetic Vulnerabilities
Source: mBio. 2021 Dec 7;12(6):e03137-21. doi: 10.1128/mBio.03137-21 (PMC8649759; doi:10.1128/mBio.03137-21)

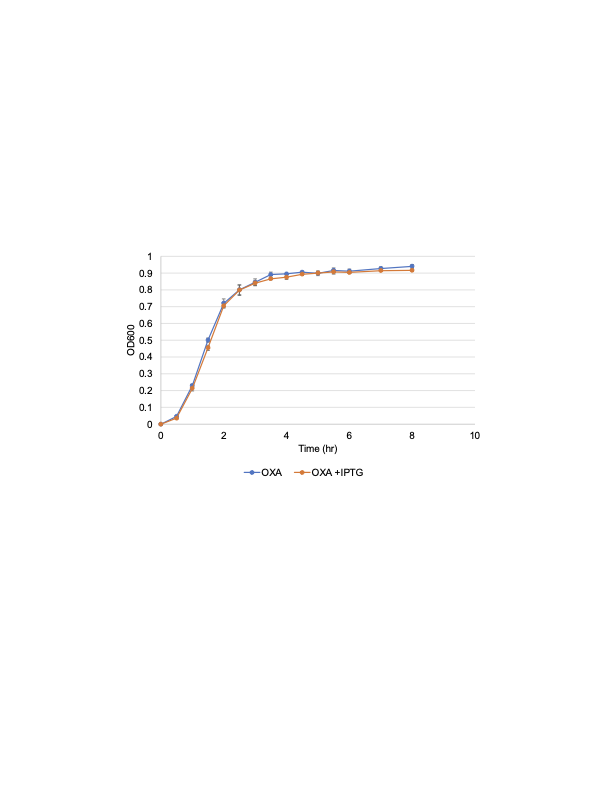

Supplement: FIG S1 [file mbio.03137-21-sf001.tif]

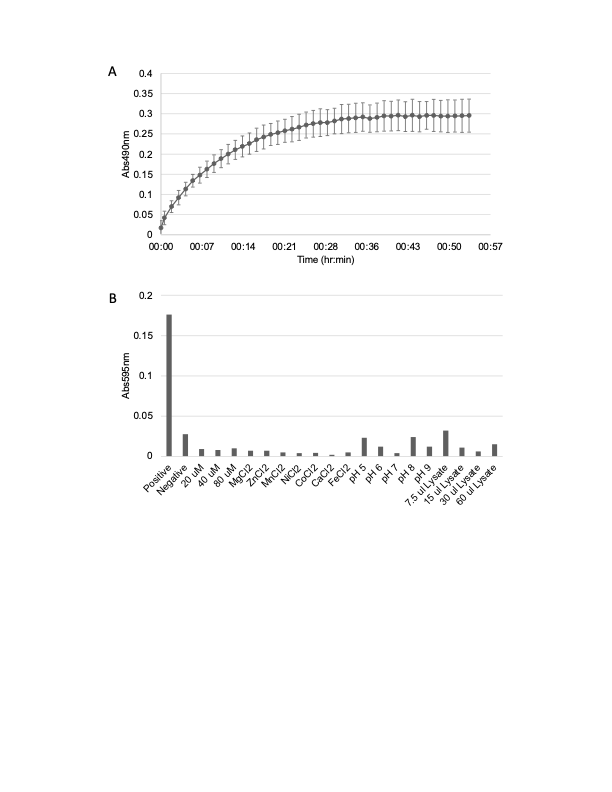

Supplement: FIG S2 [file mbio.03137-21-sf002.tif]
