# Supplementary material for: OXA-23 β-Lactamase Overexpression in Acinetobacter baumannii Drives Physiological Changes Resulting in New Genetic Vulnerabilities
Source: mBio. 2021 Dec 7;12(6):e03137-21. doi: 10.1128/mBio.03137-21 (PMC8649759; doi:10.1128/mBio.03137-21)
Supplement: TABLE S2 [file mbio.03137-21-st002.docx]

Table S2. Identification of muropeptides in mutanolysin digests from wild-type

and OXA-23 expressing *Acinetobacter baumannii* ATCC17978
