# Supplementary material for: OXA-23 β-Lactamase Overexpression in Acinetobacter baumannii Drives Physiological Changes Resulting in New Genetic Vulnerabilities
Source: mBio. 2021 Dec 7;12(6):e03137-21. doi: 10.1128/mBio.03137-21 (PMC8649759; doi:10.1128/mBio.03137-21)
Supplement: TABLE S3 [file mbio.03137-21-st003.docx]

| Table S3. Bacterial strains used in this study. | |  |
| --- | --- | --- |
|  |  |  |
| Name | Relevant Genotype | Reference |
| *E. coli* |  |  |
| EC100D | F− *mcrA* Δ(*mrr*-*hsdRMS-mcrBC*) ϕ80d*lacZ*ΔM15 Δ*lacX74 recA1 endA1 araD139* Δ(*ara-leu*)*7697 galU galK* λ− *rpsL nupG pir*+ (DHFR) | Epicentre |
|  |  |  |
| *A. baumannii* |  |  |
| ATCC 17978 | Wild type, β-lactam sensitive | 1 |
| ATCC 17978 Tn7:: *bla*_OXA-23_ |  | This Study |
| ATCC 17978 Tn7:: *bla*_OXA-23_ (S79A) |  | This Study |
| A1S_1185::kan | ATCC 17978 Tn7::OXA A1S_1185::EZTn5<KAN-2> | This Study |
| A1S_0408::kan | ATCC 17978 Tn7::OXA A1S_0408::EZTn5<KAN-2> | This Study |
| ZipA::kan | ATCC 17978 Tn7::OXA ZipA::EZTn5<KAN-2> | This Study |
| YDA007 | ATCC 17978 Tn7::dCas9 | 2 |

REFERENCES

1. Smith MG, Gianoulis TA, Pukatzki S, Mekalanos JJ, Ornston LN, Gerstein M, Snyder M. 2007. New insights into *Acinetobacter baumannii* pathogenesis revealed by high-density pyrosequencing and transposon mutagenesis. Genes Dev 21:601–614.https://doi.org/10.1101/gad.1510307.
2. Bai J, Dai Y, Farinha A, Tang AY, Syal S, Vargas-Cuebas G, van Opijnen T, Isberg RR, Geisinger E. 2021. Essential gene analysis in *Acinetobacter baumannii* by high-density transposon mutagenesis and CRISPR interference. J Bacteriol 203:e00565-20.https://doi.org/10.1128/JB.00565-20.
