# Supplementary material for: OXA-23 β-Lactamase Overexpression in Acinetobacter baumannii Drives Physiological Changes Resulting in New Genetic Vulnerabilities
Source: mBio. 2021 Dec 7;12(6):e03137-21. doi: 10.1128/mBio.03137-21 (PMC8649759; doi:10.1128/mBio.03137-21)
Supplement: TABLE S4 [file mbio.03137-21-st004.docx]

| Table S4. Plasmids used in this study. | |  |
| --- | --- | --- |
|  |  |  |
| Name | Relevant genotype | Reference |
| pUC18-mini-Tn7-LAC-Apra | *E. coli/A. baumannii* shuttle vector, Apra^R^ | Addgene |
| pTn7::*bla*_OXA-23_ | *E. coli/A. baumannii* shuttle vector. Mini-Tn7 with IPTG inducible OXA, Apra^R^ | This Study |
| pTn7::*bla*_OXA-23_ (S79A) | *E. coli/A. baumannii* shuttle vector. Mini-Tn7 with IPTG inducible OXA-S79A, Apra^R^ | This Study |
| pWH1266 | *E. coli/A. baumannii* shuttle vector, Tc^R^ | 1 |
| pWH1266::zipA | ZipA complementation in pWH1266 | 2 |
| pQF1266-hyg | derived from pQF50, *E. coli/A. baumannii* shuttle vector, Hyg^R^ | 3 |
| pQF1266-hyg::A1S_1185 | A1S_1185 complementation in pQF1266-hyg | This Study |
| pQF1266-hyg::A1S_0408 | A1S_0408 complementation in pQF1266-hyg | This Study |
| pQF1266-hyg::*bla*_OXA-23_ | Constitutive expression of OXA-23 in pQF1266-hyg | This Study |
| pJE53 | ATc inducible non-targeting sgRNA | 4 |
| pJE53::murA-sgRNA | ATc inducible CRISPRi-mediated knockdown of MurA | This Study |

REFERENCES

1. Hunger M, Schmucker R, Kishan V, Hillen W. 1990. Analysis and nucleotide sequence of an origin of DNA replication in *Acinetobacter calcoaceticus* and its use for *Escherichia coli* shuttle plasmids. Gene 87:45–51.https://doi.org/10.1016/0378-1119(90)90494-c.
2. Knight D, Dimitrova DD, Rudin SD, Bonomo RA, Rather PN. 2016. Mutations decreasing intrinsic beta-lactam resistance are linked to cell division in the nosocomial pathogen *Acinetobacter baumannii*. Antimicrob Agents Chemother 60:3751–3758.https://doi.org/10.1128/AAC.00361-16.
3. Anderson SE, Sherman EX, Weiss DS, Rather PN. 2018. Aminoglycoside heteroresistance in *Acinetobacter baumannii* AB5075. mSphere 3:e00271-18.https://doi.org/10.1128/mSphere.00271-18.
4. Bai J, Dai Y, Farinha A, Tang AY, Syal S, Vargas-Cuebas G, van Opijnen T, Isberg RR, Geisinger E. 2021. Essential gene analysis in *Acinetobacter baumannii* by high-density transposon mutagenesis and CRISPR interference. J Bacteriol 203:e00565-20.https://doi.org/10.1128/JB.00565-20.
